# Quantitative Proteomics of Cerebrospinal Fluid in Paediatric Pneumococcal Meningitis

**DOI:** 10.1038/s41598-017-07127-6

**Published:** 2017-08-01

**Authors:** Guadalupe Gómez-Baena, Richard J. Bennett, Carmen Martínez-Rodríguez, Małgorzata Wnęk, Gavin Laing, Graeme Hickey, Lynn McLean, Robert J. Beynon, Enitan D. Carrol

**Affiliations:** 1 Centre for Proteome Research, Institute of Integrative Biology, University of Liverpool, Liverpool, L69 7ZB United Kingdom; 2 Department of Clinical Infection, Microbiology and Immunology, Institute of Infection and Global Health, University of Liverpool, Liverpool, L69 7BE United Kingdom; 3 Department of Molecular Biology and Parasitology, School of Tropical Medicine, Liverpool, L3 5QA United Kingdom; 4 Department of Biostatistics, Institute of Translational Medicine, Liverpool, L69 3BX United Kingdom

## Abstract

*Streptococcus pneumoniae* is responsible for diseases causing major global public health problems, including meningitis, pneumonia and septicaemia. Despite recent advances in antimicrobial therapy, pneumococcal meningitis remains a life-threatening disease. Furthermore, long-term sequelae are a major concern for survivors. Hence, a better understanding of the processes occurring in the central nervous system is crucial to the development of more effective management strategies. We used mass spectrometry based quantitative proteomics to identify protein changes in cerebrospinal fluid from children with *Streptococcus pneumoniae* infection, compared with children admitted to hospital with bacterial meningitis symptoms but negative diagnosis. Samples were analysed, by label free proteomics, in two independent cohorts (cohort 1: cases (n = 8) and hospital controls (n = 4); cohort 2: cases (n = 8), hospital controls (n = 8)). Over 200 human proteins were differentially expressed in each cohort, of which 65% were common to both. Proteins involved in the immune response and exosome signalling were significantly enriched in the infected samples. For a subset of proteins derived from the proteome analysis, we corroborated the proteomics data in a third cohort (hospital controls (n = 15), healthy controls (n = 5), cases (n = 20)) by automated quantitative western blotting, with excellent agreement with our proteomics findings. Proteomics data are available via ProteomeXchange with identifier PXD004219.

## Introduction

Acute bacterial meningitis (ABM) is usually fatal without treatment, and prompt and accurate diagnosis coupled with timely administration of appropriate parenteral antibiotics, are essential to save lives^1–3^. The three vaccine-preventable organisms causing ABM in low to middle income countries (LMICs) are: *Haemophilus influenzae* type b, *Streptococcus pneumoniae* and *Neisseria meningitidis*.

*Streptococcus pneumoniae* infection is a leading cause of pneumonia, meningitis and septicemia worldwide, and results in approximately 1 million deaths in children under the age of 5 years annually^4^. Pneumococcal meningitis is a life-threatening disease with poor prognosis associated with neurologic complications and a high case-fatality ratio in sub-Saharan Africa, which is further increased by HIV co-infection^5^. The intricate host inflammatory response is associated with neuronal and vascular injury, even after cerebrospinal fluid (CSF) sterilization with antibiotics. Adjunctive new therapies for bacterial meningitis to date have not shown any conclusive benefit, prompting the need for an improved understanding of key mechanisms that might reveal potential new diagnostic and therapeutic targets^6^.

In pneumococcal meningitis, 2D-electrophoresis based proteomics of CSF has demonstrated an exacerbated host response, which participates in brain damage, leading to the nomination of several proteins as potential biomarkers for validation^7–9^. However, 2D electrophoresis can lack sensitivity compared with other proteomic approaches such as bottom up proteomics. Several studies have shown the potential of shotgun proteomics in providing information on the pathophysiology of neurological disorders through the differential identification and quantification of proteins in CSF from controls and cases studies^10–14^.

The aim of the present work was to identify protein changes in CSF associated with paediatric *Streptococcus pneumoniae* meningitis, as this pathogen is the commonest cause of ABM in children in those areas with a high incidence of HIV infection. We utilized label-free quantitative mass spectrometry based proteomics to define qualitative and quantitative differences in the proteins present in CSF, derived either from host (human) or from the pathogen, *Streptococcus pneumoniae*. We used as controls, CSF from children admitted to hospital with symptoms of ABM but who were negative after diagnostic tests. For a subset of proteins, we confirmed the proteomic data using an orthogonal technique, automated quantitative western blotting. Protein changes detected are robust and consistent among cohorts. To our knowledge, this is the first detailed global discovery-shotgun quantitative proteomic study reported for pneumococcal meningitis in children. Proteomic data are available via ProteomeXchange with identifier PXD004219.

## Results

### Identification of CSF proteins

**Figure 1:**
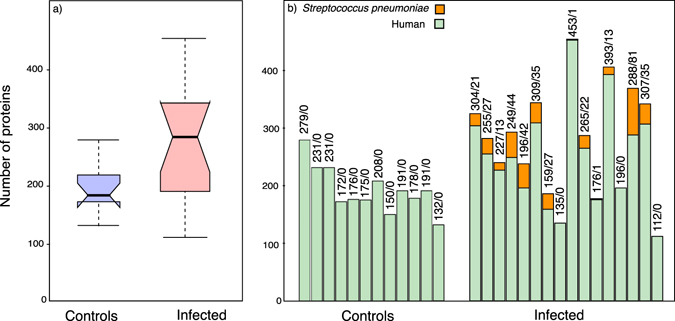
Proteome profiles of control and infected CSF samples. For each sample, protein discovery analysis was performed on a fixed volume of CSF from control (n = 12) and infected individuals (n = 16). (Panel a) Box plots analysing the number of protein groups obtained in control (blue) and infected (red) samples. Top and bottom of the box represent the 75% (Q3) and 25% (Q1) percentile, the line inside the box represents the median and whiskers define 1.5 times the interquartile range from the box. (Panel b) Number of host and pathogen proteins identified per sample. Each column expresses the number of human proteins identified in the sample (green) and the number of *S. pneumoniae* proteins (orange).

To profile the protein changes in infected CSF, we used a fixed volume of fluid (20 µl) from 28 CSF samples (hospital controls = 12; *S. pneumoniae* positive (SPP) = 16); overall experimental plan is in Supplementary Fig. 1 and clinical data from patients in Supplementary Table 1). Proteins in the CSF samples were denatured, digested with trypsin and the resultant tryptic peptides were resolved by high-resolution reversed-phase chromatography prior to tandem mass spectrometry of individual peptides. The raw tandem MS files were used to search an annotated database of human proteins (version 20151209; 20,187 entries) and a database of *S. pneumococcus* proteins (version 20151209; 2,030 entries). To ensure confidence in proteins listed here, we adopted a rigorous filtering criterion, restricting the protein list to those that were identified with at least two unique peptides after applying a 1% FDR at the peptide level. More proteins were identified in SPP samples by a considerable margin (Fig. 1a). The total number of proteins that were identified varied considerably, irrespective of infection status, the lowest being 112 (SPP) and the highest being 454 (also an SPP sample) (Fig. 1b). For the overall data set, over 190 protein groups were common to all control and SPP samples whereas 111 were identified exclusively in control and 411 protein families were only identified in SPP samples. Our intention in this study was to define the proteome changes using a fixed volume of CSF, rationalising that any future test of infection status would adopt the same protocol. It follows that the protein input into each analysis would be reflected in the number of protein groups identified, but this parameter is also conditioned by the dynamic range of protein expression; high abundance proteins can prevent selection of low abundance peptides for fragmentation. This is supported by the label-free proteome analysis below.

All the SPP CSF samples had been identified as positive for *S. pneumoniae* and all control samples were diagnosed as negative for this pathogen. We assessed whether *S. pneumoniae* proteins exceeded the limit of detection such as to permit full discrimination between control and SPP samples (Fig. 1b). No *S. pneumoniae* proteins could be detected in any control sample, but in SPP samples, the average number of *S. pneumoniae* proteins was 30 ± 6 (mean ± SEM, n = 13, excluding the SPP samples in which no *S. penumoniae* proteins were detected). In thirteen out of sixteen SPP samples, it was possible to identify proteins from the bacterial pathogen *S. pneumoniae*. As expected, these were largely abundant soluble components such as ribosomal proteins and glycolytic enzymes, but also some membrane-associated proteins such as the cell-wall associated serine proteinase PrtA (UniprotKB Q8DQP7), ABC-transporter substrate-binding protein-oligopeptide transport (UniprotKB Q8DNI1), ABC-transporter substrate-binding protein sugar transport (UniprotKB Q8DNU8) and manganese ABC transporter substrate-binding lipoprotein (UniprotKB P0A4G3).

Compared to the number of host protein identifications, relatively few pathogen proteins were identified. However, this should not occasion surprise. The typical bacterial load in these samples was of the order of 10,000 organisms per mL of CSF (Supplementary Table 1). At 100 fg of protein per typical bacterial cell^15^ this would lead to a bacterial protein load of 10^4^ * 100 fg = 10^6^ fg per mL, or 1 ng bacterial protein per mL of CSF, against a host background of 0.1 to 5 mg/mL. In other words, the bacterial load typically expanded the CSF protein abundance by no more than 0.001% to 0.1% of the total pool. When the bacterial protein pool is distributed over multiple proteins, the infectious agent would introduce new proteins that are largely below limits of global proteomics detectability and which would be obscured by host proteins, other than the most abundant. It is thus unsurprising that so few pathogen proteins were detectable using this approach. More advanced assays (based on highly specific antibodies or using selected reaction monitoring) might provide enough sensitivity for unambiguous diagnosis, but relatively simple label-free methods are likely to struggle.

**Figure 2:**
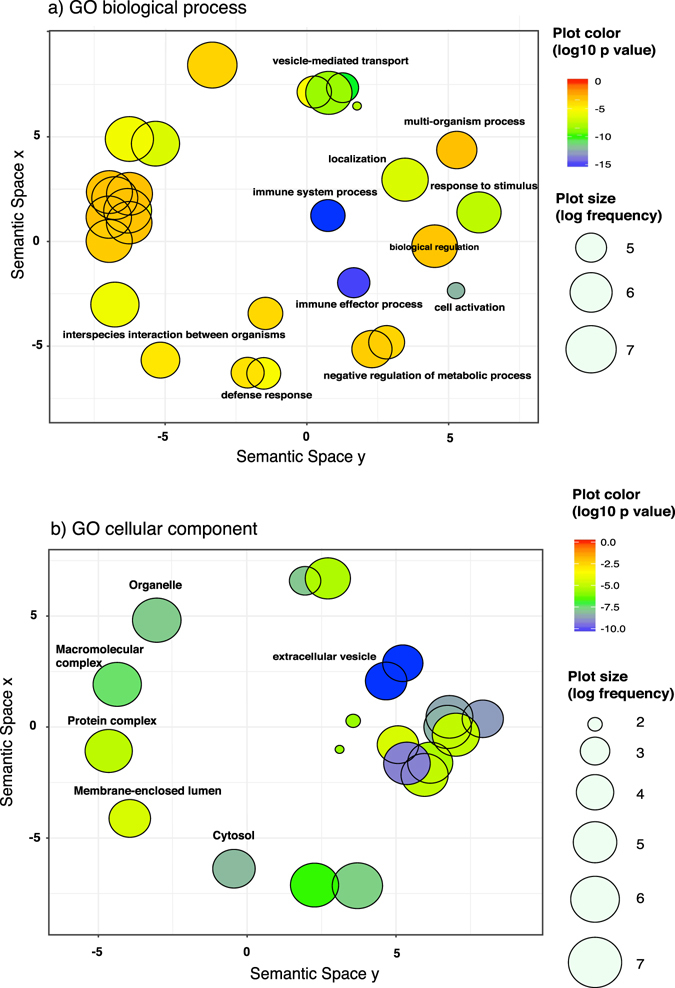
Gene ontology enrichment analysis. GO enrichment analysis of host (human) proteins was performed using GOrilla^16^ and summarized and visualized as scatter plot using REvigo^17^. (Panel a) shows a scatter plot generated in REvigo with the “GO biological terms” clusters remaining after redundancy reduction. (Panel b) shows a scatter plot generated in REvigo with the “GO cellular components clusters” remaining after redundancy reduction. In REvigo the x and y coordinates are derived from a multidimensional scaling to a matrix of the GO terms semantic similarity^17^, in such a way that similar terms are located close in the plot. Only those terms with dispensability value equal 0.00 and p value < 0.001 are labelled. Plot colour indicates the enrichment log p value range and the plot size indicates the frequency of the GO term in the database. More general terms are showed with larger symbols.

We performed enrichment analysis for Gene Ontology (GO) terms of host (human) proteins using GOrilla^16^. This approach allows us to obtain a global picture highlighting processes triggered during infection. The proteins identified in the control samples were used as the background for analysis in GOrilla. The analysis of terms in the “GO biological processes” ontology showed significant enrichment (p value < 0.001, q value < 0.01) in SPP samples for terms related to the immune system, defence response, and response to external biotic stimulus (Supplementary Fig. 2). Significantly enriched terms were summarized and clustered using REvigo^17^ (Fig. 2a) showing significant enrichment for cluster containing terms as immune system process, immune effector process or defence response. Increased neutrophil-derived proteins reflect the immune reaction to bacterial infection. A typical finding in bacterial meningitis is an increased WBC count in CSF, which is one of the traditional diagnostic criteria^18^.

Analysis of terms in the “cellular component” ontology pointed to “exosomes” and “extracellular vesicles” enriched in SPP samples (Fig. 2b, Supplementary Fig. 3). Exosomes are small extracellular vesicles that have been postulated to play a role in intercellular communication, modulation of the immune response and antigen presentation^19^. Dendritic cells derived exosomes activate T cells^20, 21^. More specifically, exosomes released from bone marrow derived murine dendritic cells, treated with the capsular polysaccharide 14 (Cps14) from *S. pneumoniae*, can induce humoral responses^22^. Our data highlight the involvement of exosomes in the pathogenesis of pneumococcal meningitis. The biological function of exosomes in the progression of the disease is still unknown. Exosomes have been described as important factors in the antigen presentation to T-cells but also potentially spreading infectious particles and worsening the symptoms by enhancing inflammatory response. Therefore, several studies are targeting exosomes as potential therapeutic targets for neurological inflammatory diseases and drug delivery particles^23, 24^.

### Label-free quantification of CSF proteins

It is possible to use information on peptide signal intensity from the protein identification analyses to derive a measure of abundance of the proteins in the samples. This approach, of label-free quantification, is particularly appropriate for the measurement of relative quantification and the comparison of patient and control samples. Samples were analysed in two independent cohorts (cohort 1: cases (n = 8) and hospital controls (n = 4); cohort 2: cases (n = 8), hospital controls (n = 8)). Label-free quantification revealed 214 protein groups that were significantly up- or down-regulated in the first cohort (Supplementary Table 2) and 234 protein groups in the second cohort (Supplementary Table 3), using the criteria of a minimum two-fold change, p value < 0.05, q value < 0.05 and quantification based on two or more unique peptides for quantification. When the two cohorts were compared, the dataset reduced to 134 host (human) and six *Streptococcus* protein groups that were common to the two cohorts (Supplementary Table 4). Proteins families sharing the same anchor protein were grouped.

**Figure 3:**
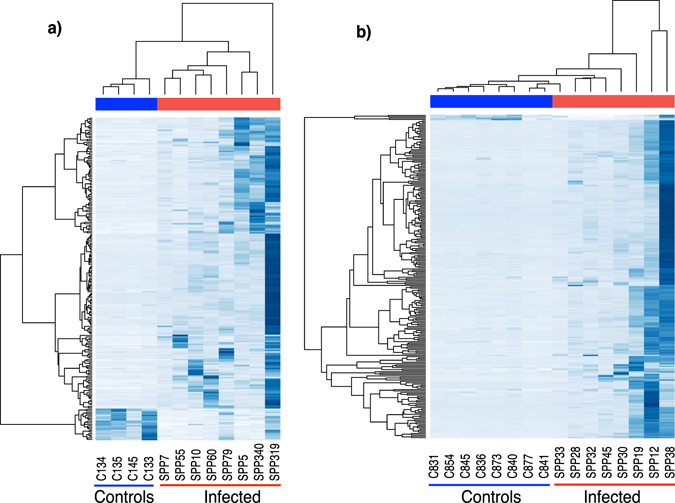
Heat maps and cluster analysis of the quantification of proteins calculated by label free approach. The overall profile of proteins that were analysed in two separate cohorts (a and b panels) of patient and control samples was expressed as a heat map, normalizing the proteome profiles for each sample by calculating the z-score on proteins. Unsupervised clustering analysis was used to segregate the samples.

The overall profile of proteins was visualised in heat map format, normalising protein expression by z-score across proteins, emphasising the breadth and abundance of the proteome profiles for each sample (Fig. 3). Clustering analysis for both cohorts confirmed clear differences between SPP samples and controls. Notwithstanding a consistent pattern of changed protein expression, there was considerable sample to sample variation, reflecting the natural variance in the protein concentration and complexity in the samples.

**Figure 4:**
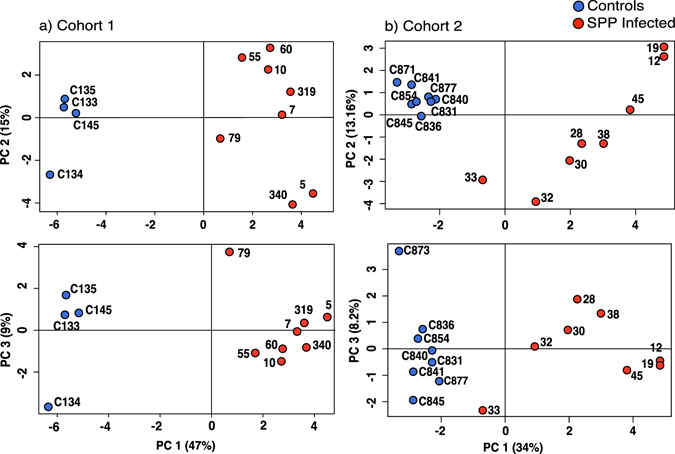
Principal component analysis of CSF proteomes. The quantitative proteome profiles obtained by label-free analysis using Progenesis QI were used to inform a principal components analysis in R. To correct for loading variance, each log protein summed intensity was normalised and expressed as z-score within the CSF sample. (Panel a) shows the result for the first cohort and (Panel b) for the second cohort. Control samples are defined by blue symbols, infected samples by red symbols.

SPP samples contained on average two orders of magnitude higher overall protein than the control samples (Supplementary Fig. 4). Since protein concentrations were higher in SPP samples than in controls, but highly variable, and we used a fixed volume of CSF per digestion, variation in host and pathogen protein levels is to be expected. Our focus was the pattern of proteins, and as such, we corrected for different protein loading, expressing each log protein summed intensity as a z-score. When log protein abundances, z-scored corrected, were compared using principal components analysis there was clear resolution of infected and non-infected samples (Fig. 4).

**Figure 5:**
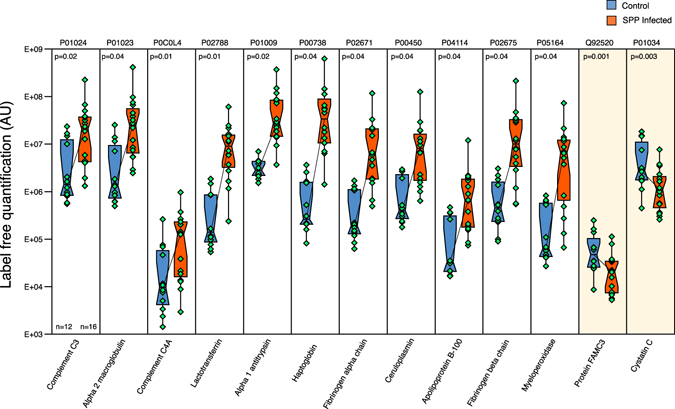
Label-free quantification of confidently identified and quantified proteins. Whiskers indicate the 95% and 5% boundaries of the data and the horizontal bar within the box is the median. Control samples are blue and SPP are red. Yellow shading is used to highlight the two proteins present at lower levels in SPP. Proteins are defined by their UniProtKB ID **P01024**: Complement C3, **P01023**: Alpha-2-macroglobulin, **P0C0L4**: Complement C4-A, **P02788**: Lactotransferrin, **P01009**: Alpha-1-antitrypsin, **P00738**: Haptoglobin, **P02671**: Fibrinogen alpha-chain, **P00450**: Ceruloplasmin, **P04114**: Apolipoprotein B-100, **P02675**: Fibrinogen beta chain, **P05164**: Myeloperoxidase, **Q92520**: Protein FAM3C, **P01034**: Cystatin-C. Probabilities were calculated using the t-test function in R and corrected for multiple testing by Benjamini and Hochberg method. Corrected p-values are shown in the panels. Individual data points are shown for controls (n = 12) and SPP (n = 16) samples.

The changes in protein abundance were dramatic (up to 500-fold change, Supplementary Tables 2 and 3), indicative of a major shift in the protein complement in CSF. Although virtually all proteins were up-regulated in SPP samples, some proteins were down-regulated. Figure 5 illustrates the magnitude of these changes for the top up-regulated and down-regulated human proteins with the highest confidence score in Progenesis QI.

### Quantitative automated western blotting

A subset of proteins was selected based on confidence of protein identification, the magnitude of change by label-free proteomics, extent of protein coverage and the putative role in the pathology of the pneumococcal meningitis. These were: S100 A9, myeloperoxidase, cathelicidin, ceruloplasmin and cystatin C. Protein S100 A9 (UniprotKB P06702) is an EF-hand Ca^2+^ binding protein of the S100 family that is abundant in the cytoplasm of several cells, including phagocytes. It forms a heterodimer, with S100 A8 (UniprotKB P05109), known as calprotectin, which promotes leukocyte recruitment and phagocytosis^25^. S100 A8 and A9 were identified in seven and six out of eight SPP samples respectively, and quantification showed up-regulation in both cohorts of samples. Myeloperoxidase (UniprotKB P05164) is a major component of neutrophil granules and it is responsible of part of their antimicrobial activity^26, 27^. This protein was identified in all infected samples and up-regulated in both cohorts. Cathelicidin antimicrobial peptide (UniprotKB P49913) is part of the innate immune response and is expressed in neutrophils and other cells including cells ﻿in the CNS. This protein is up-regulated in SPP samples and has been recently reported as putative biomarker of ABM in children^28^. Ceruloplasmin (UniprotKB P00450) is a copper-containing enzyme that also plays a role in iron metabolism and antioxidant defence in serum and CSF^29^. Lastly, we selected cystatin C (UniprotKB P01034), a cysteine protease inhibitor belonging to the cystatin family that has a protective role in neurological diseases^30, 31^. By contrast with the other proteins, it was down-regulated in the SPP samples.

**Figure 6:**
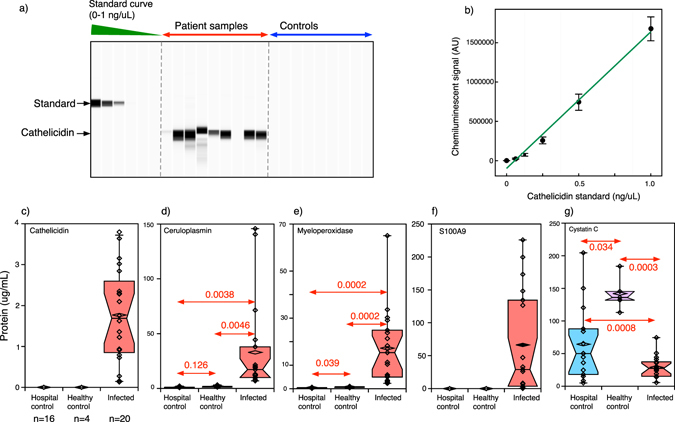
Automated western blotting of selected CSF proteins. Five proteins (cathelicidin (panel c), ceruloplasmin (panel d), myeloperoxidase (panel e), protein S100A9 (panel f) and cystatin C (panel g) were quantified in a larger cohort (n = 40, 15 hospital controls, 5 healthy controls and 20 SPP patients) using automated capillary western blotting (*Wes, ProteinSimple, CA, USA*). A typical ‘pseudo-gel image is shown in panel a, highlighting the position of the GST:cathelicidin fusion standard protein and the native cathelicidin the corresponding standard curve (panel b). Purified recombinant proteins were used as calibration standards for all five proteins. Panels c to g are the summarised quantitative western blot data for cathelicidin, ceruloplasmin, myeloperoxidase, S110A9 and cystatin C respectively. Dots show individual results for SPP samples (red) and hospital controls (blue) and healthy controls (purple). Top and bottom of the box represent the 75% (Q3) and 25% (Q1) percentile, the band inside the box is the median and whiskers extend to 1.5 times the interquartile range from the box. Points outside the box are outliers. For each protein, t-tests were used to assess the differences between samples.

These proteins were quantified in a larger cohort of additional samples (n = 40, 20 controls and 20 SPP). Significant changes in protein abundances were observed between the SPP samples and two sets of controls, hospital and commercial controls (p < 0.05) in the same direction predicted by the proteomic approach (Fig. 6). Principal component analysis on the absolute quantification data was able to discriminate between SPP and control samples (not shown). Additionally, we demonstrated good correlation between the concentration of host proteins measured using quantitative automated western blot and CSF WBC for ceruloplasmin, myeloperoxidase and S100A9, but not cathelicidin or cystatin C (Supplementary Table 5).

## Discussion

Normal and disease specific components in CSF reflect pathological processes occurring in the CNS. Quantitative proteomics allows the capture of multi-dimensional patterns and pathways, as those occurring in complex biological processes such as meningitis^32^. For this reason, quantitative proteomics is of considerable value in the generation of a host response protein signature from which insight into pathogenesis can be achieved.

The pathogenesis of ABM has multiple stages, including mucosal colonization, invasion of the intravascular space, bacteraemia and disruption of the blood-brain-barrier (BBB) (for a review^33, 34^). The elevated host proteins in CSF may have originated at CNS or may be a result of BBB disruption. Once the bacteria enter the CNS, they multiply and induce the release of pro-inflammatory compounds that cause pleocytosis and increased BBB permeability. As our analysis shows, this mechanism is reflected in the protein signature of SPP CSF, notably through enrichment in neutrophil and plasma derived proteins. Neutrophil-derived proteins, as neutrophil gelatinase-associated lipocalin (UniprotKB P80188), neutrophil collagenase (UniprotKB P22894), neutrophil elastase (UniprotKB P08246), myeloperoxidase (UniprotKB P05164), azurocidin (UniprotKB P20160), and cathelicidin antimicrobial peptide (UniprotKB P49913), are up regulated in SPP samples. Antimicrobial activity is also part of the SPP signature. Lysozyme (UniprotKB P61626) and lactotransferrin (UniprotKB P02788) are elevated in SPP samples. Lysozyme participated in the destruction of *S. pneumoniae* ^35^ and lactotransferrin binds pneumococcal surface protein A having an important role in bactericidal function^36^.

Hemopexin (UniprotKB P02790) and haptoglobin (UniprotKB P00738) are also up-regulated. These two plasma proteins play a key role in detoxifying CSF from hemoglobin and the free heme group in processes involving extravascular hemolysis. Free heme-iron participates in oxygen radical reactions that covalently modify proteins, lipids, carbohydrates and nucleotides leading to tissue damage^37, 38^.

One important component of the immune system is the complement activation pathway. The complement system is involved in phagocytosis and the assembly of the membrane attack complex, which promotes cell lysis by forming pores in the membrane of gram-negative bacteria^39^. From our data and those from previous studies on pneumococcal meningitis, complement C3 is increased in SPP samples^8, 9, 40^ and here, we demonstrate an increase in concentration of other components of the complement system from the classical pathway (complement C4-A (UniprotKB P0C0L4), Complement C1r subcomponent (UniprotKB P00736), complement factor I (UniprotKB P05156), Complement C2 (UniprotKB P06681)) and the alternative pathway (complement factor B (UniprotKB P00751) and complement factor H (UniprotKB P08603)). Further, the membrane attack complex (complement C6 (UniprotKB P13671), C7 (UniprotKB P10643) and C9 (UniprotKB P02748)) is also more abundant in SPP samples.

The host response to infection includes the up regulation of members of the coagulation cascade and anticoagulation processes. Dysregulation of the fibrinolytic agents is the cause of cerebrovascular complications in patients with bacterial meningitis^41^. C-reactive protein (UniprotKB P02741) and fibrinogen beta chain (UniprotKB P02675) are increased in SPP samples as previously reported^8, 9, 40, 42^. Our analysis also shows increased levels of fibrinogen alpha chain (UniprotKB P02671) and gamma chain (UniprotKB P02679) chains. Anticoagulant proteins are also higher in SPP samples including C-reactive protein (UniprotKB P02741), antithrombin III (UniprotKB P01008) plama protease C1 inhibitor (UniprotKB P05155) and plasminogen (UniprotKB P00747).

It is also noteworthy that a number of proteases and proteases inhibitors were elevated in SPP samples. Matrix metalloproteinase 9 (MMP9; UniprotKB P14780) is up regulated in our analysis. Concentration of this protein correlates with poor clinical outcome in patients with bacterial meningitis and it is associated with neurological sequelae^43, 44^, since high concentrations of MMP9 promote brain damage^45^.

Our proteomic approach offers improved understanding of the pathogenic process involving neurological damage and cell death. Neuronal expression of vimentin (UniprotKB P08670), a cytoskeletal protein, is seen in Alzheimer disease, and there is evidence that neurons express vimentin as a damage-response mechanism^46^. This protein was elevated in our study consistent with the pathophysiology of pneumococcal meningitis promoting cell death and tissue destruction. Cathepsin B is also up regulated and its activity has been related with brain injury in a murine model of pneumococcal meningitis^47^. It has been reported that Cathepsin B is up regulated by neutrophil elastase, a protease also elevated in SPP samples^48^. In animal models, it has been reported that cellular damage occurs via caspase cascade, however, no caspases have been identified in our data, nor in a previous study on pneumococcal meningitis^7^.

Host-pathogen interaction proteins, as vitronectin (UniprotKB P04004), are increased in SPP samples. Vitronectin plays a significant role in neutrophil cell migration, tissue repair and regulation of the membrane attack complex^49, 50^. However, *S pneumoniae* utilises vitronectin for effective adhesion to host cells and subsequent internalisation^49, 50^. Therefore, vitronectin plays a role in bacterial pathogenesis by helping pathogens evade the host response and could be a potential target for drug development. Besides, several *S. pneumoniae* membrane proteins were identified suggesting these as potential therapeutic targets. These proteins are crucial in pathogenesis, participating in host-pathogen interaction during infection and playing a central function in nutrient uptake. Surface-exposed proteins linked to glucose metabolism, such as glyceraldehyde-3-phosphate dehydrogenase (UniprotKB Q8CWN6) and enolase (UniprotKB Q8DPS0), could prove to be worthy candidates as therapeutic targets. We selected a panel of proteins for further analysis by western blotting. These were: S100 A9, myeloperoxidase, cathelicidin, ceruloplasmin and cystatin C. Significant changes were obtained in the same trend anticipated by proteomics. Moreover, we demonstrated significant correlations between three of the proteins selected and CSF WBC, suggesting a concerted cellular host response against the infecting pathogen (Supplementary Table 5).

To our knowledge, this is the first shotgun proteomic survey reported in patients suffering from pneumococcal meningitis. Our study provides a global view of the complex processes occurring in the CNS during *Streptococcus pneumoniae* infection. We also report a comprehensive list of proteins that significantly increase during infection, which improves the understanding of key mechanisms and might reveal potential new diagnostic and therapeutic targets. Further, we confirmed some of the LC-MS/MS data by a quantiatively robust orthogonal method.

## Methods

### Ethical statement

Ethical approval for this study was granted from The College of Medicine Research Committee, Malawi and The Liverpool School of Tropical Medicine Local Research Ethics Committee. All methods were performed in accordance with relevant guidelines and regulations. Parents or guardians gave written informed consent for children to enter the study. The study was part of a larger prospective observational study investigating the genetic susceptibility to invasive pneumococcal disease in Malawian children^51^. This study was conducted at Queen Elizabeth Central Hospital in Blantyre (Malawi) between April 2004 and October 2006. Details of the enrolment criteria, standard operating procedures and management protocols have been described elsewhere^52^. Clinical data and outcomes for these patients are compiled in Supplementary Table 1. Confirmation of ABM was defined by a child presenting symptoms of ABM, a CSF cell count > 10/mm^3^ and one of the following tests for *Streptococcus pneumoniae* positive: CSF culture, Gram stain, polysaccharide antigen or PCR. All samples were cultured on sheep blood and chocolate agar for 48 h under aerobic and microaerophilic conditions. Bacteria were identified using standard methods^53^. Pneumococcal bacterial DNA was amplified and quantified using a real-time PCR assay as previously described^52^. All CSF samples were spun down within 2 h of collection and the supernatant fraction was frozen within 4 h of collection, and stored at −80 °C until analysis.

### In-solution digestion

CSF (20 μL) was incubated with RapiGest SF surfactant (Waters Corporation, Milford, MA) at a final concentration of 0.05% (w/v) for 10 min at 80 °C in 25 mM ammonium bicarbonate. Samples were then reduced with 3 mM DTT for 10 min at 60 °C, followed by alkylation with 9 mM iodoacetamide for 30 min in the dark at room temperature. Finally, trypsin was added and incubated overnight at 37 °C. To stop the proteolytic reaction and to inactivate and precipitate the detergent, trifluoroacetic acid (final concentration 0.5% (v/v)) was added, followed by incubation for 45 min at 37 °C. Samples were centrifuged at 13,000 g for 15 min and the supernatant peptide fraction analysed by LC-MS/MS.

### Liquid Chromatography-Tandem Mass Spectrometry (LC-MS/MS) analysis

LC-MS/MS analysis was performed on a nanoAcquity chromatography system (*Waters Corporation*, Milford, MA) coupled to a LTQ-Orbitrap Velos (*Thermo Fisher Scientific*, Waltham, MA). CSF digests were trapped onto a Symmetry C18 precolumn (180 μm id, 20 mm long, 5 μm particles) (*Waters Corporation*, Milford, MA) over 3 min, at a flow rate of 25 μL/min in 2% (v/v) ACN /0.1% (v/v) formic acid. Bound peptides were resolved on a nanoAcquity UPLC C18 column (75 μm id, 150 mm long, 3 μm particles) at 300 nL/min over a 120 min linear gradient from 3 to 85% (v/v) ACN in 0.1% v/v formic acid. The instrument was operated in data-dependent acquisition mode. A fixed volume (0.5 μL) of digestion was analysed and the 20 most intense multiply charged ions were sequentially fragmented at 35% of normalized collision energy. Precursors selected were dynamically excluded for 20 s.

### Database search parameters and acceptance criteria for identification

Raw data were converted into a single *.mgf format peaklist file by Proteome Discoverer 1.1 (Thermo Fisher Scientific, Waltham, MA) using default parameters. Independent *.mgf files for each sample were searched against a database composed of reviewed entries of Human Uniprot database (version 20151209; 20,187 entries) and Streptococcus pneumoniae reference strain ATCC BAA-255/R6 (version 20151209; 2,030 entries) with MASCOT search engine (version 2.5.1, Matrix Science), using trypsin as specific enzyme, carbamidomethylation of cysteine as fixed modification, methionine oxidation as variable modification and one trypsin missed cleavage, a mass tolerance of 10 ppm for precursors and 0.6 Da for fragment ions. The false discovery rate (FDR) was calculated using the decoy database tool in MASCOT. Only those proteins identified by at least 2 significant peptides, and at a FDR <1% were accepted. The minimum list of proteins explaining the set of peptides identified was built using the Report builder in MASCOT. The mass spectrometry proteomics data have been deposited to the ProteomeXchange Consortium via the PRIDE^54^ partner repository with the dataset identifier PXD004219 and 10.6019/PXD004219.

### Label-free protein quantification and analysis of differential protein expression

Proteins were quantified using Progenesis QI software v2.0 (Waters Corporation, Milford, MA). Quantification was based on unique peptides, raw abundances and non-conflicting features. The abundance of a peptide was calculated from the peak area and the protein abundance was calculated from the sum of all unique peptide abundances for a specific protein across each sample. Features with positive charge states between 2 and 5, and three or more isotopic peaks were taken to further analysis. Different biological samples were grouped as control or infected. A merged peaklist generated by Progenesis QI was searched against the database described in the section above, using MASCOT search engine (version 2.5.1, Matrix Science) and the same search parameters. A cut off score of 20 was applied after manually evaluating the quality of the lowest scored peptides. Proteins containing similar peptides were grouped into families. The criteria to consider a protein to be significantly up- or down-regulated were: a fold change between groups greater than a 2-fold using at least 2 unique peptides, p value < 0.05 and q value < 0.05, calculated in Progenesis QI.

### Quantitative western blotting

Automatic western blots were performed using a Wes automated system (ProteinSimple, California, USA). Purified recombinant proteins were used as calibration standards. Serial dilutions of both sample and standard were used to determine the linear dynamic range of the assay. Additionally, the optimal concentration of each antibody for use in the Wes system was determined, as this can differ from that used in traditional western blot. Samples (20 SPP, 15 hospital controls and 5 healthy controls) were mixed with a 5x sample buffer containing SDS, DTT and fluorescent molecular weight standards and heated at 95 °C for 5 min and then, loaded onto a plate prefilled with stacking and separation matrices, along with blocking and wash buffers, antibody solutions and detection reagents. Default settings were used for the analysis.

### Pathway analysis

Enrichment analysis for Gene Ontology (GO) terms of host (human) proteins identified in the present study was perfomed using GOrilla^16^. The list of proteins identified in the control samples was used as background for analysis (two-unranked list of genes). P value cut off was set up at 0.001 and FDR q value at 0.01. Gorilla database used was last updated on Jan 21, 2017. Significant enriched terms were summarized using REvigo. For Go biological process a 0.5 similarity was allowed (small list), while a 0.7 was allowed for cellular component (medium list).

### Statistical analysis

Data were analysed and visualised using Aabel(*Gigawiz software*, http://www.gigawiz.com/) and R (v.3.2) (http://www.R-project.org/).

## Electronic supplementary material

Supplementary information

Supplementary Table 2

Supplementary Table 3

Supplementary Table 4
