# Supplementary material for: Quantitative Proteomics of Cerebrospinal Fluid in Paediatric Pneumococcal Meningitis
Source: Sci Rep. 2017 Aug 1;7:7042. doi: 10.1038/s41598-017-07127-6 (PMC5539295; doi:10.1038/s41598-017-07127-6)
Supplement: Supplementary file 1 — Supplementary information [file 41598_2017_7127_MOESM1_ESM.pdf]

SUPPLEMENTARY INFORMATION

Quantitative Proteomics of Cerebrospinal Fluid in Paediatric Pneumococcal Meningitis

Guadalupe Gómez-Baena, Richard J. Bennett, Carmen Martínez-Rodríguez, Małgorzata Wnęk, Gavin Laing, Graeme Hickey, Lynn McLean, Robert J. Beynon, Enitan D. Carrol

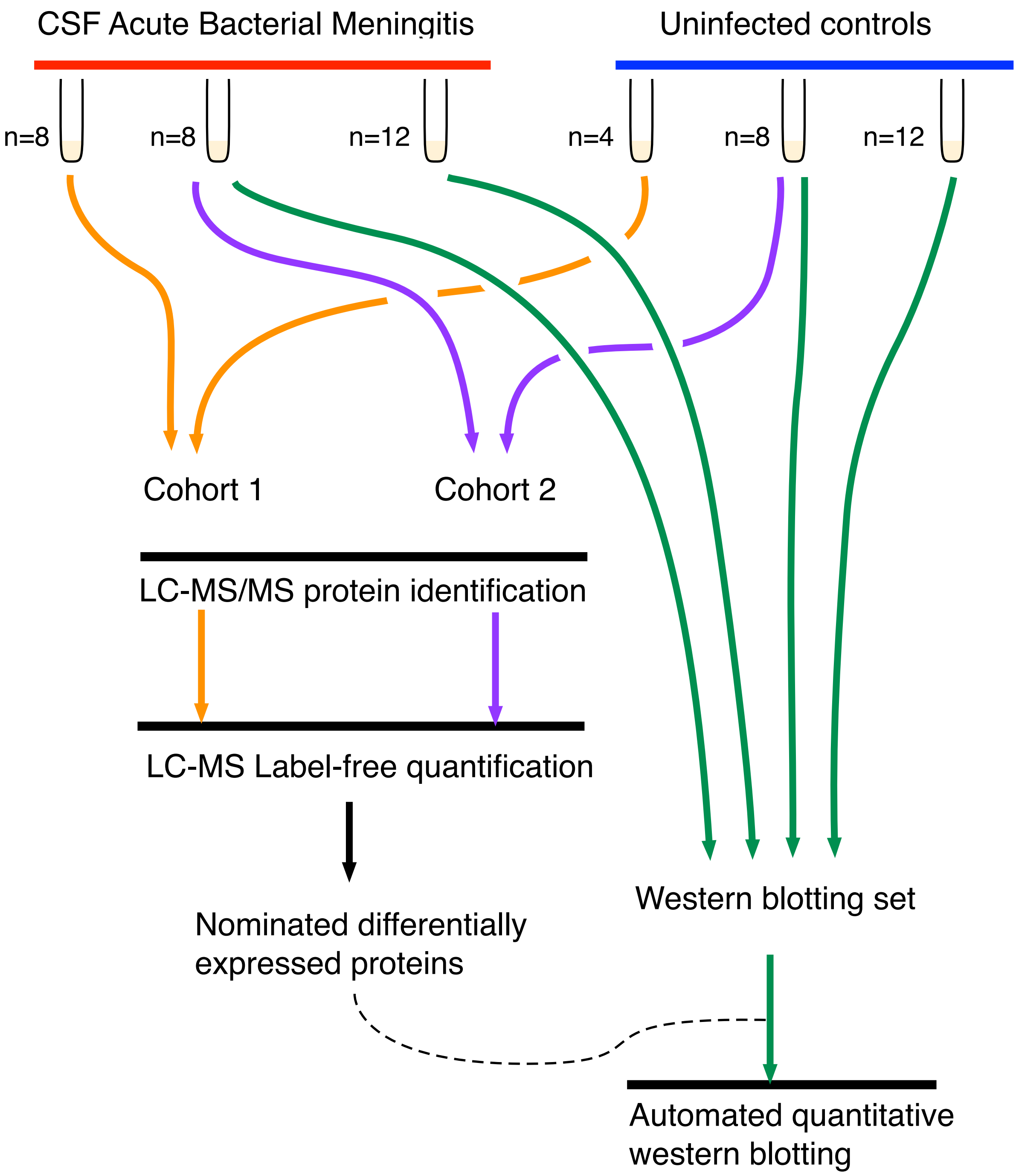

Supplementary Figure 1: Experimental design

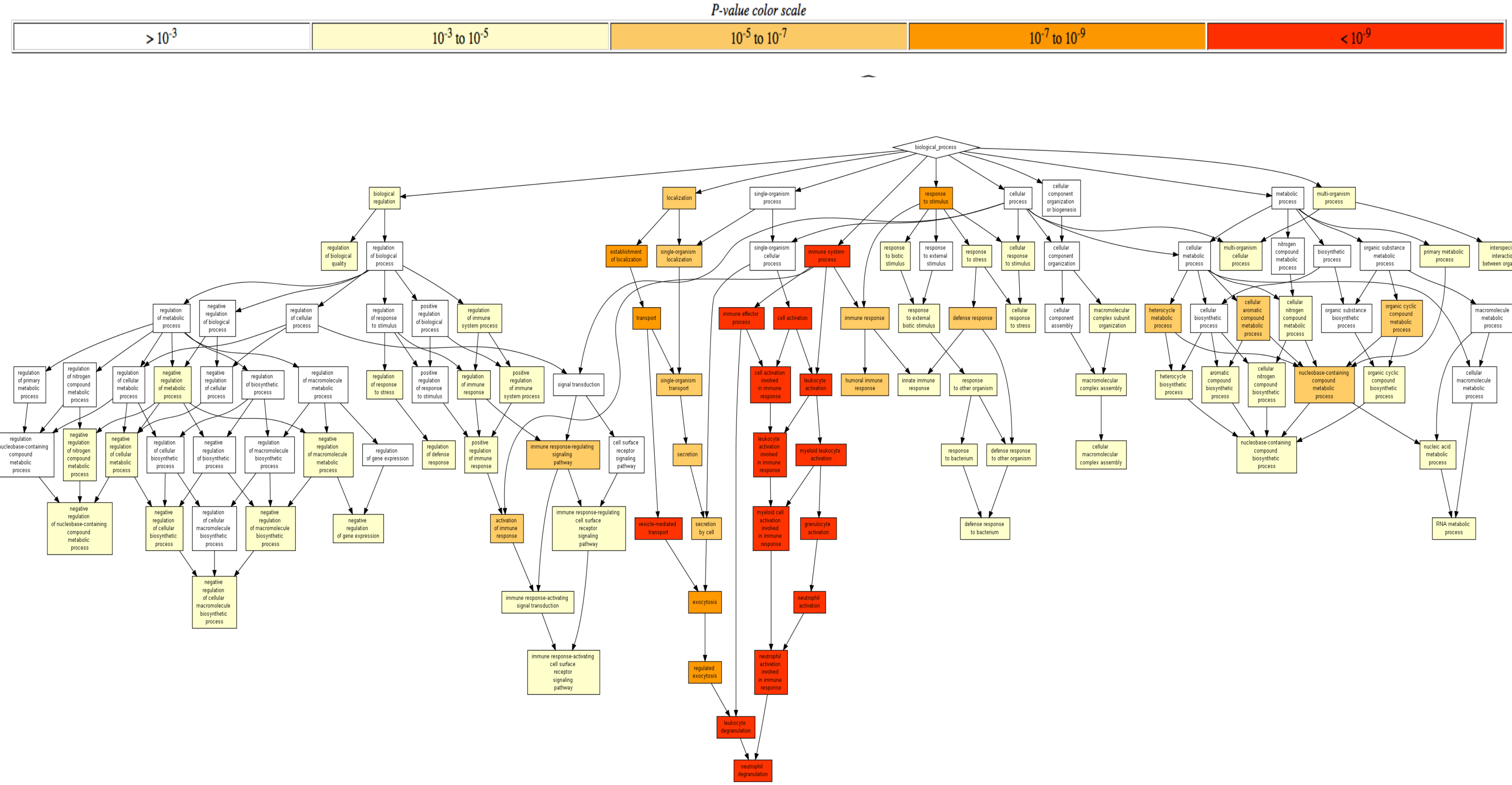

Supplementary Figure 2. GO ontology enrichment analysis: GO biological process. Enrichment analysis for biological process ontology was performed using GOrilla (http://cbl-gorilla.cs.technion.ac.il). The list of proteins identified in control samples was used as background list, and the list of proteins identified in SPP samples as target. The system recognized 758 out of 904 terms entered and 583 of these terms were associated with a GO term.

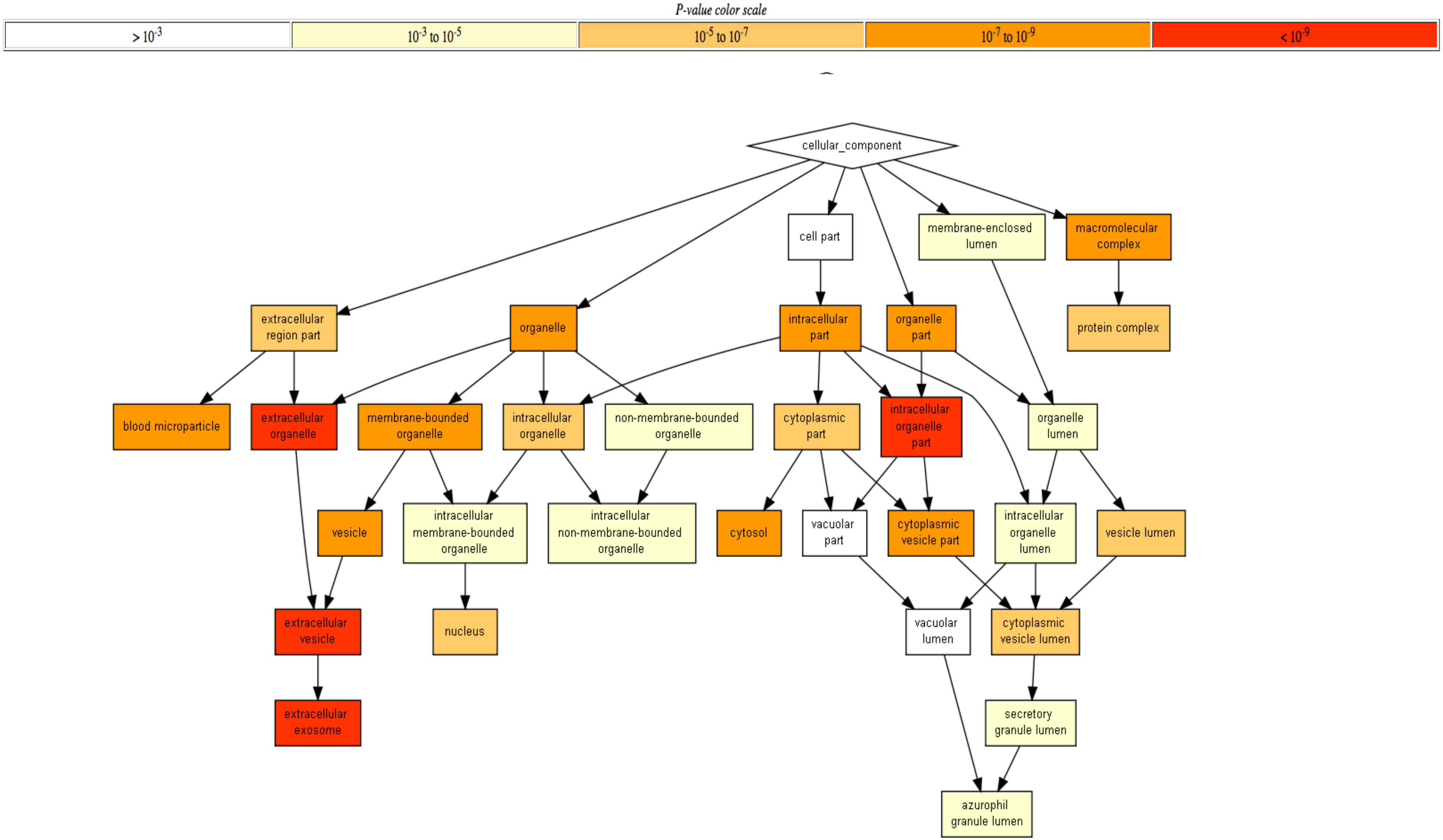

Supplementary Figure 3. GO ontology enrichment analysis: GO cellular component. Enrichment analysis for Cellular components ontology was performed using GOrilla (http://cbl-gorilla.cs.technion.ac.il). The list of proteins identified in control samples was used as background list, and the list of proteins identified in SPP samples as target. The system recognized 758 out of 904 terms entered and 583 of these terms were associated with a GO term.

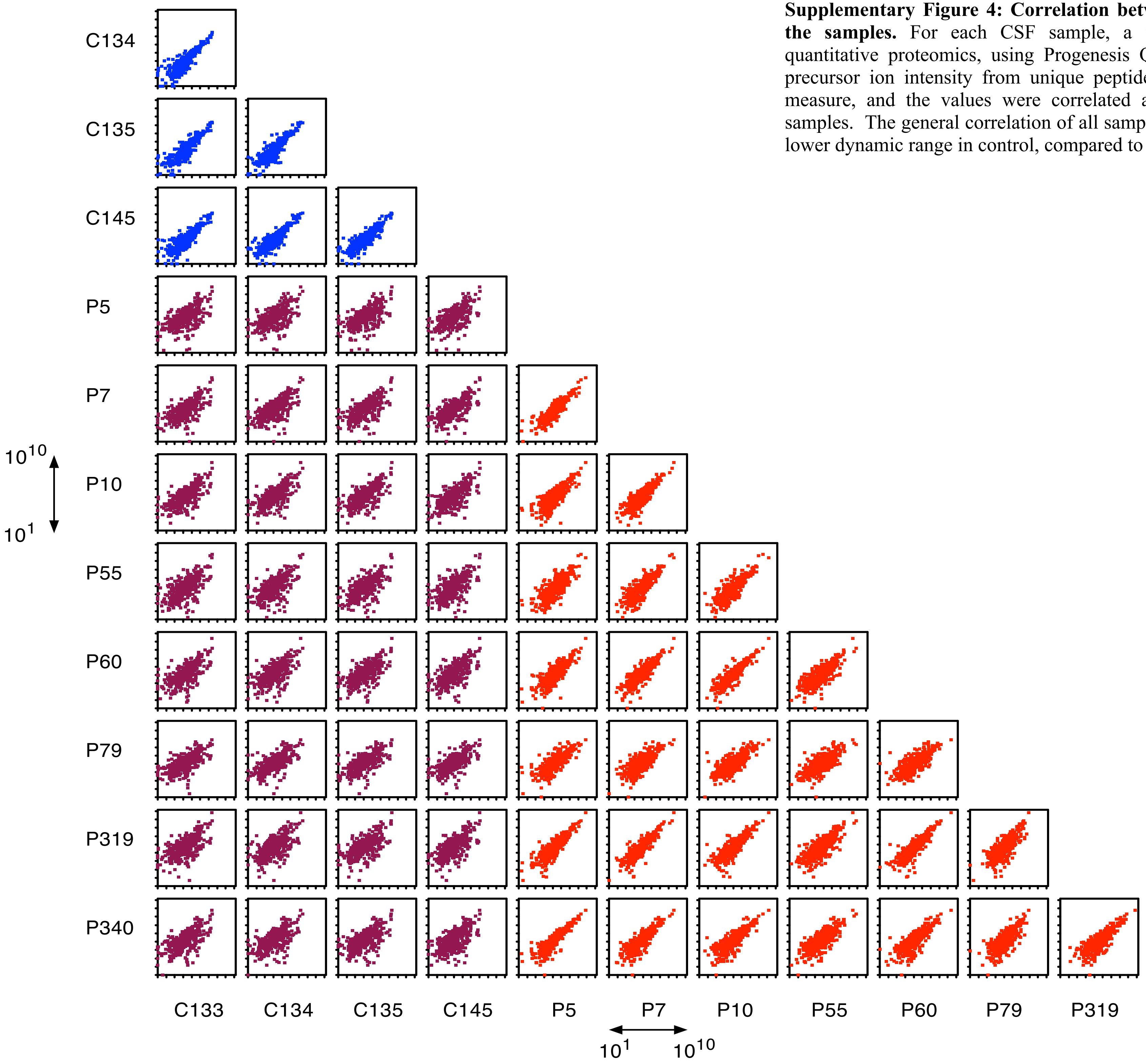

Supplementary Figure 4: Correlation between abundances of proteins identified in the samples. For each CSF sample, a fixed volume was analysed by label-free quantitative proteomics, using Progenesis QI software. For each protein, the summed precursor ion intensity from unique peptides was used to obtain a protein abundance measure, and the values were correlated across all four controls, and all eight SPP samples. The general correlation of all samples is apparent from the comparison, as is the lower dynamic range in control, compared to infected samples.

SPP SAMPLE	Protein	Age (years)	Sex	WBC (cells/mm^3)	Bacterial load (copies/mL)	Blantyre Coma Score	Outcome
79	4+	1.08	F	6080	1890	1	Alive
60	4+	0.25	M	975	11126	3	Alive
55	4+	5.83	F	3120	11.78	4	Alive
10	4+	0.42	M	ND	1028	3	Alive
7	ND	5.84	F	ND	730	0	Died
5	ND	3.25	F	ND	95790	2	Alive
319	4+	0.67	F	2480	1050718690	3	Died
340	4+	13.0	F	10480	152249	5	Alive
12	3	0.42	F	2480	25620	2	Died
19	4+	4.0	M	3600	7050	1	Died
28	4+	0.33	F	105	45640	5	Alive
30	4+	9.0	M	235	725	5	Alive
32	4+	1.17	M	12	6157	3	Died
33	4+	0.5	M	2480	5152	4	Alive
38	4+	0.33	F	3440	ND	2	Died
45	4+	1.0	F	1638	607	5	Alive

Supplementary Table 1: Patient and sample characteristics of bacterial meningitis cases

BLANTYRE COMA SCORE [1]	SCORE
**Best motor response**	
Localizes painful stimulus	2
Withdraws limb from pain	1
Non-specific or absent response	0
**Verbal response**	
Appropriate cry or normal speech	2
Moan or inappropriate cry	1
None	0
**Eye movement**	
Directed (e.g follows mother's face)	1
Not directed	0
Maximum score 5, Minimum score 0	

1. Molyneux ME, Taylor TE, Wirima JJ, Borgstein A: **Clinical features and prognostic indicators in paediatric cerebral malaria: a study of 131 comatose Malawian children**. *Q J Med* 1989, **71**(265):441-459.

Supplemetary Table 5: Correlation coefficients (Spearman’s rho) between CSF host proteins and CSF white cell count

**Biomarker**	CSF White Cell Count
S100A8/9	0.48<br>p=0.04
Myeloperoxidase	0.80<br>p<0.0005
Cathelicidin	0.19<br>p=NS
Ceruloplasmin	0.73<br>p<0.0005
Cystatin C	-0.09<br>p=NS

NS=not significant
